# Prioritization of oligogenic variant combinations in whole exomes

**DOI:** 10.1093/bioinformatics/btae184

**Published:** 2024-04-11

**Authors:** Barbara Gravel, Alexandre Renaux, Sofia Papadimitriou, Guillaume Smits, Ann Nowé, Tom Lenaerts

**Affiliations:** Interuniversity Institute of Bioinformatics in Brussels, Université Libre de Bruxelles-Vrije Universiteit Brussel, 1050 Brussels, Belgium; Department of Computer Science, Machine Learning Group, Université Libre de Bruxelles, 1050 Brussels, Belgium; Department of Computer Science, Artificial Intelligence Laboratory, Vrije Universiteit Brussels, 1050 Brussels, Belgium; Interuniversity Institute of Bioinformatics in Brussels, Université Libre de Bruxelles-Vrije Universiteit Brussel, 1050 Brussels, Belgium; Department of Computer Science, Machine Learning Group, Université Libre de Bruxelles, 1050 Brussels, Belgium; Department of Computer Science, Artificial Intelligence Laboratory, Vrije Universiteit Brussels, 1050 Brussels, Belgium; Interuniversity Institute of Bioinformatics in Brussels, Université Libre de Bruxelles-Vrije Universiteit Brussel, 1050 Brussels, Belgium; Department of Computer Science, Machine Learning Group, Université Libre de Bruxelles, 1050 Brussels, Belgium; Brussels Interuniversity Genomics High Throughput core (BRIGHTcore), UZ Brussel, Vrije Universiteit Brussel (VUB) - Université Libre de Bruxelles (ULB), 1090 Brussels, Belgium; Interuniversity Institute of Bioinformatics in Brussels, Université Libre de Bruxelles-Vrije Universiteit Brussel, 1050 Brussels, Belgium; Center of Human Genetics, Hôpital Erasme, Hôpital Universitaire de Bruxelles, Université Libre de Bruxelles, 1070 Brussels, Belgium; Interuniversity Institute of Bioinformatics in Brussels, Université Libre de Bruxelles-Vrije Universiteit Brussel, 1050 Brussels, Belgium; Department of Computer Science, Artificial Intelligence Laboratory, Vrije Universiteit Brussels, 1050 Brussels, Belgium; Interuniversity Institute of Bioinformatics in Brussels, Université Libre de Bruxelles-Vrije Universiteit Brussel, 1050 Brussels, Belgium; Department of Computer Science, Machine Learning Group, Université Libre de Bruxelles, 1050 Brussels, Belgium; Department of Computer Science, Artificial Intelligence Laboratory, Vrije Universiteit Brussels, 1050 Brussels, Belgium

## Abstract

**Motivation:**

Whole exome sequencing (WES) has emerged as a powerful tool for genetic research, enabling the collection of a tremendous amount of data about human genetic variation. However, properly identifying which variants are causative of a genetic disease remains an important challenge, often due to the number of variants that need to be screened. Expanding the screening to combinations of variants in two or more genes, as would be required under the oligogenic inheritance model, simply blows this problem out of proportion.

**Results:**

We present here the High-throughput oligogenic prioritizer (Hop), a novel prioritization method that uses direct oligogenic information at the variant, gene and gene pair level to detect digenic variant combinations in WES data. This method leverages information from a knowledge graph, together with specialized pathogenicity predictions in order to effectively rank variant combinations based on how likely they are to explain the patient’s phenotype. The performance of Hop is evaluated in cross-validation on 36 120 synthetic exomes for training and 14 280 additional synthetic exomes for independent testing. Whereas the known pathogenic variant combinations are found in the top 20 in approximately 60% of the cross-validation exomes, 71% are found in the same ranking range when considering the independent set. These results provide a significant improvement over alternative approaches that depend simply on a monogenic assessment of pathogenicity, including early attempts for digenic ranking using monogenic pathogenicity scores.

**Availability and implementation:**

Hop is available at https://github.com/oligogenic/HOP.

## 1 Introduction

Identifying the exact genetic causes of a patient’s disease remains an important challenge in medical genetics. Despite the advances brought by next-generation sequencing technologies and Whole-Exome Sequencing (WES), which, since 2012, enabled the identification of >100 novel disease–genes per year (Boycott *et al.* 2017), many genetic disorders are linked with a low percentage of confirmed diagnoses (Stavropoulos *et al.* 2016, Shickh *et al.* 2021). The identification of a precise genetic diagnosis is hindered by the phenotypic variability, genetic heterogeneity and reduced penetrance associated with such diseases (Cooper *et al.* 2013, Woodward *et al.* 2022). Obtaining an exact genetic diagnosis is important in order to tailor the patients’ treatments based on their molecular diagnosis, develop new types of treatment, as well as bring an end to their diagnostic odysseys (Graungaard and Skov 2007, Splinter *et al.* 2018).

Many different computational approaches have been developed to try to tackle such issues and to aid clinical researchers in prioritizing the genetic variants that are more likely to be causative of a patient’s disease. The most successful algorithms integrate information about the patient’s phenotype, which is collected and standardized in the Human Phenotype Ontology (HPO) (Köhler *et al.* 2021), together with variant data. These “phenotype-driven” approaches to prioritization are based on the idea that diseases with similar symptoms are likely to be caused by similar genes (Smedley and Robinson 2015). Examples of such tools, which have been recently reviewed (Yuan *et al.* 2022), include Exomiser (Robinson *et al.* 2014), Phevor (Singleton *et al.* 2014), and AMELIE (Birgmeier *et al.* 2020). These tools score genetic variants using both phenotype-relevance measures, which assess how likely it is for a gene to be involved in the phenotype of the patient, and pathogenicity measures, which evaluate the potential of a variant or gene to be involved in a disease mechanism. Their scores are then integrated to obtain a final ranking (Smedley and Robinson 2015, Yuan *et al.* 2022).

Phenotype-relevance scoring relies on the guilt-by-association principle and on previous knowledge about gene–phenotype associations, in order to identify new genes with similar profiles to disease genes (Zolotareva and Kleine 2019). Network-based methods are being increasingly used for that purpose as they rely on the intuitive aggregation of different sources of information into a single network. Indeed, different studies have shown that disease genes tend to be organized in modules, which means that novel gene-disease associations can be discovered by looking for genes that are closely related to known disease–genes in different types of networks (Agrawal *et al.* 2018, Buphamalai *et al.* 2021). Based on this hypothesis, successful prioritization methods were developed that use the proximity to known disease genes in networks as a measure for prioritizing variants or genes (Köhler *et al.* 2008, Lysenko *et al.* 2017, Peng *et al.* 2017, Zhang *et al.* 2018).

Notwithstanding their success, the aforementioned prioritization tools have been tailored towards the identification of causes for Mendelian (or monogenic) diseases, which directly limits their relevance. Indeed, an increasing number of diseases have been shown to have a more complex genetic aetiology (Robinson and Katsanis 2010, Deltas 2017), and where the involved mutations can have different properties than the monogenic ones. With the release of a first curated collection of known digenic disease instances, i.e. the Digenic Diseases Database or DIDA (Gazzo *et al.* 2016), a first attempt at oligogenic prioritization was made (Boudellioua *et al.* 2018). However, this tool was trained to make monogenic predictions and then tested on its capacity to also prioritize variant combinations. The latter required genes to be connected in a protein-protein interaction (PPI) network, implying that unconnected genes in this network [which represent 88/189, i.e. 47% of the combinations tested in Boudellioua *et al.* (2018)] would not be ranked.

The recent publication of OLIDA (Nachtegael *et al.* 2022), which collects information on >1000 variant combinations reported to be involved in oligogenic diseases, provided the grounds for further developments in the field, as for instance the recent release of VarCoPP2.0 (Versbraegen *et al.* 2023) (see Supplementary Section S1 for details on this method). This novel machine learning classifier improves VarCoPP (Papadimitriou *et al.* 2019), by achieving higher accuracy and drastically reducing the computational time required for predictions. Although it performs well in assessing the potential pathogenicity of a small set of pre-filtered variant pairs (like in a gene panel), its effectiveness on a patient’s complete WES data is hindered by its 5% false-positive rate. The huge number of variant combinations that are produced from a single VCF file may translate in thousands of variant combinations predicted as potentially disease-causing, making their further analysis impossible without some stringent filtering. There is thus a strong need to automatically filter and prioritize the variant combinations in relation to the phenotype or disease that is being investigated so that users can focus on the most relevant results.

We present here the High-throughput oligogenic prioritizer (Hop), an innovative prioritization tool that utilizes explicitly oligogenic information at the variant and gene level to prioritize digenic variant combinations. By combining the results produced by VarCoPP2.0 with background information about diseases, Hop effectively ranks, in a patient’s WES data, variant combinations suspected to be causative of the patient’s disease. The method takes as input a patient’s exome, together with information on the patient’s disease to generate the ranking. Variant combinations in gene pairs are scored with a pathogenicity score and a disease-relevance score, obtained by propagating the information on the patient’s disease in a knowledge graph specifically tailored to the study of human genetic diseases. These two scores are then combined to obtain a final ranking. We demonstrate the effectiveness of this method on synthetic exomes and show its success in prioritizing artificially inserted known disease-causing combinations. We show that Hop provides a complementary insight in a patient’s WES data to what monogenic state-of-the-art prioritization methods may discern and that it performs significantly better than early attempts to digenic prioritization.

## 2 Materials and methods

### 2.1 Datasets

The method takes as input genotype data, in the form of a Variant Call Format (VCF) file, and information about the patient’s disease, in the form of a combination of HPO terms, describing the patient’s phenotype, and a gene panel associated with the disease, or either one of them if only one is available. Since WES data of patients with an oligogenic disease for which the combination underlying the disease was known are not publicly available, we generated synthetic datasets as follows.

#### 2.1.1 Genotype data

The VCF files were generated by inserting oligogenic combinations from the OLIDA database (Nachtegael *et al.* 2022) in exomes from the 1000 genomes project (1KGP) (Auton *et al.* 2015) and the UK10K project (Walter *et al.* 2015).

OLIDA (v3 May 2023) combinations involving variants in two genes were selected and further filtered based on their confidence scores, considering only combinations with FINALmeta score of at least 1. This resulted in the inclusion of 420 combinations to insert in 1KGP (and UK10K) exomes. Since some of these combinations were used as training instances for the VarCoPP2.0 model, the model used in Hop required retraining when determining the performance of Hop as this overlap could bias otherwise the results (see Section 2.2 and Supplementary Section S1). For the purpose of this paper, we divided the 420 OLIDA combinations in a “training set”, which comprises only the combinations that were used as training instances for VarCoPP2.0 (301 combinations), and a “testing set”, which consists of the remaining (unseen) combinations that are used for independent validation of Hop (119 combinations).

We selected 100 individuals at random from the 1KGP project, with 20 individuals from each continent, as exome templates for our synthetic exomes. Since VarCoPP2.0 was partly trained on 1KGP data, we also included 20 individuals selected at random from the UK10K ALSPAC cohort as additional, completely independent, exome templates. In total, our prioritization method is thus be evaluated on 36 120 synthetic exomes containing the training instances (120 templates × 301 combinations) and 14 280 synthetic exomes containing the testing instances (120 templates × 119 combinations). The identifiers of the OLIDA combinations, and of the 1KGP and UK10K individuals are available in Supplementary Tables S3 and S4.

The variants of these synthetic exomes were then filtered based on Minor Allele Frequency (MAF) and variant position criteria, following what was done for the training of the VarCoPP2.0 model (Versbraegen *et al.* 2023). These variant filters are based on the characteristics of the variants found to be causative of an oligogenic disease as reported in OLIDA. More specifically, we removed variants with MAF >3.5%, synonymous variants that are further than 195 nucleotides from exon edges, and intronic variants.

#### 2.1.2 Disease-related data

When the information was available, each synthetic patient was annotated with both phenotypic data, describing the patient’s symptoms, and a gene panel, associated with the patient’s disease. The contribution of both types of information for prioritization is analysed in this work.

Phenotypic data is encoded as terms from the Human Phenotype Ontology (HPO) (Köhler *et al.* 2021). In order to obtain accurate phenotypic annotation that would mimic case descriptions, the HPO terms are extracted from the articles that described the OLIDA combinations. For each OLIDA combination in our training and test sets, we went back to the article that identified the combinations, and annotated each described case manually and with the help of the Text Annotator tool from the Monarch Initiative (McMurry *et al.* 2016, Shefchek *et al.* 2020). Each OLIDA combination, and subsequent synthetic patient carrying that combination, is thus annotated with HPO terms corresponding to the phenotype caused by that combination. The annotations are available in Supplementary Table S3.

Each synthetic patient is assigned a disease based on the disease linked to the OLIDA combination carried by that patient. OLIDA links each combination to diseases from the Orphanet and/or OMIM databases. To annotate synthetic patients with the relevant gene panels, an extensive search was conducted in the Genomics England PanelApp (Martin *et al*. 2019) for Orphanet and/or OMIM diseases associated with the combinations in OLIDA. The associations between diseases and gene panel were manually reviewed to ensure accuracy in matching. The gene panels associated with each OLIDA combination are also made available in Supplementary Table S3 for transparency. Note that some diseases lack associated gene panels, i.e. 98/420 combinations did not have gene-panel information for prioritization (Supplementary Table S1) and can thus only rely on the HPO information for prioritization.

### 2.2 Scoring variant combinations pathogenicity

For pathogenicity scoring, Hop uses the VarCoPP2.0 predictor (Versbraegen *et al.* 2023), a machine learning model directly predicting the pathogenicity of variant combination in gene pairs (see Supplementary Material, Supplementary Section S1). Hop generates all possible variant combinations in two genes, with a maximum number of 2 variants per gene, from the filtered VCF file. These combinations are then annotated with the 15 biological features used by VarCoPP2.0, and the model prediction, which assigns a prediction score between 0 and 1, which is interpreted as the probability that the variant combination may be pathogenic (Versbraegen *et al.* 2023). This VarCoPP2.0 prediction score will be referred to as the *pathogenicity score* (*PS*) in Hop.

In the case of the exomes where the combinations inserted belonged to the OLIDA “training set” (see Section 2.1.1 for details), the predictive VarCoPP2.0 model used to score the combinations is evaluated using a custom cross-validation procedure: the training set is divided in 10-folds of equal size, ensuring that all variant combinations in the same gene-pair are part of the same fold. For each fold (referred to as test-fold), a model with the same structure as the VarCoPP2.0 predictor is then trained on the training set generated by the other nine folds, which is in turn used to predict the exomes where the variant combinations belonging to the test-fold were inserted. Given this setting, the model used to predict the pathogenicity of the variant combinations in a synthetic exome has never seen the actual pathogenic OLIDA combination in its training set, and therefore allows for a correct estimation of the performance of the predictor. All information pertaining to the training of Hop is summarized in the DOME Supplementary Table S2 (Walsh *et al*. 2021).

### 2.3 Scoring gene pairs’ relevance to the disease

In order to score the relevance of a gene pair for a disease, we used a network propagation strategy, more precisely a random walk with restart algorithm, leveraging the connectivity in a heterogeneous network.

#### 2.3.1 Heterogeneous network as background information

The background biological knowledge is obtained from BOCK, a knowledge graph or heterogeneous network containing a variety of molecular and disease information resources (Renaux *et al.* 2023). This resource aggregates heterogeneous data from 12 biological databases and annotates, using a specific node type, whether gene combinations have been implicated in a digenic disease as reported in OLIDA (see Supplementary Fig. S1 as well as Supplementary Section S2).

For the research here, the oligogenic interaction nodes and their links to the diseases and genes nodes are removed (as this is what the method aims to detect). Moreover, as random walks will be performed on this knowledge graph we do not take into account the edges weights, which results in considering the knowledge graph as an unweighted, multiplex-heterogeneous network with 8 layers (see Supplementary Fig. S1).

#### 2.3.2 Random walk with restart algorithm for disease-relevance scoring

Random walk with restart (RWR) is a type of network propagation algorithm that disseminates the information from a set of user-defined seed nodes through a network, and outputs a score for each node in the network, which can be interpreted as a measure of proximity of the node to the set of seeds. This algorithm is known to take into account both local and global network topology when performing the propagation (Cowen *et al.* 2017). The main intuition behind applying an RWR algorithm for disease–gene prioritization is the “guilt-by-association” principle, which is based on the fact that genes involved in the same disease are usually close in biological networks (Goh *et al.* 2007, Menche *et al.* 2015).

The RWR is formally defined as:

(1) $$p^{t\;+\;1}=(1\;-\;r)Wp^{t}\;+\;rp^{0}$$

where *W* is the column-normalized adjacency matrix of the graph and *p^t^* is a vector in which the *i*th element represents the probability of being at node *i* at time *t*, *r* is the restart probability, a parameter set between 0 and 1, which controls the “spread” of the walk on the network. If the restart parameter is high, the walker will often go back to the seed nodes, and only nodes close to the seeds will be traversed, while if the restart parameter is closer to 0, the walker will be able to reach nodes further from the initial nodes. Different restart parameter values were investigated in order to analyze the influence of this parameter on the ranking (see Supplementary Fig. S2) and the final value is set to 0.3 based on that analysis. The *p*^0^ vector represents the initial starting point of the walk, and is here constructed by setting equal probabilities to all seed nodes, while reaching a sum of 1, which is equivalent to having the random walker start randomly from any of the seed nodes.

**Figure 1.**
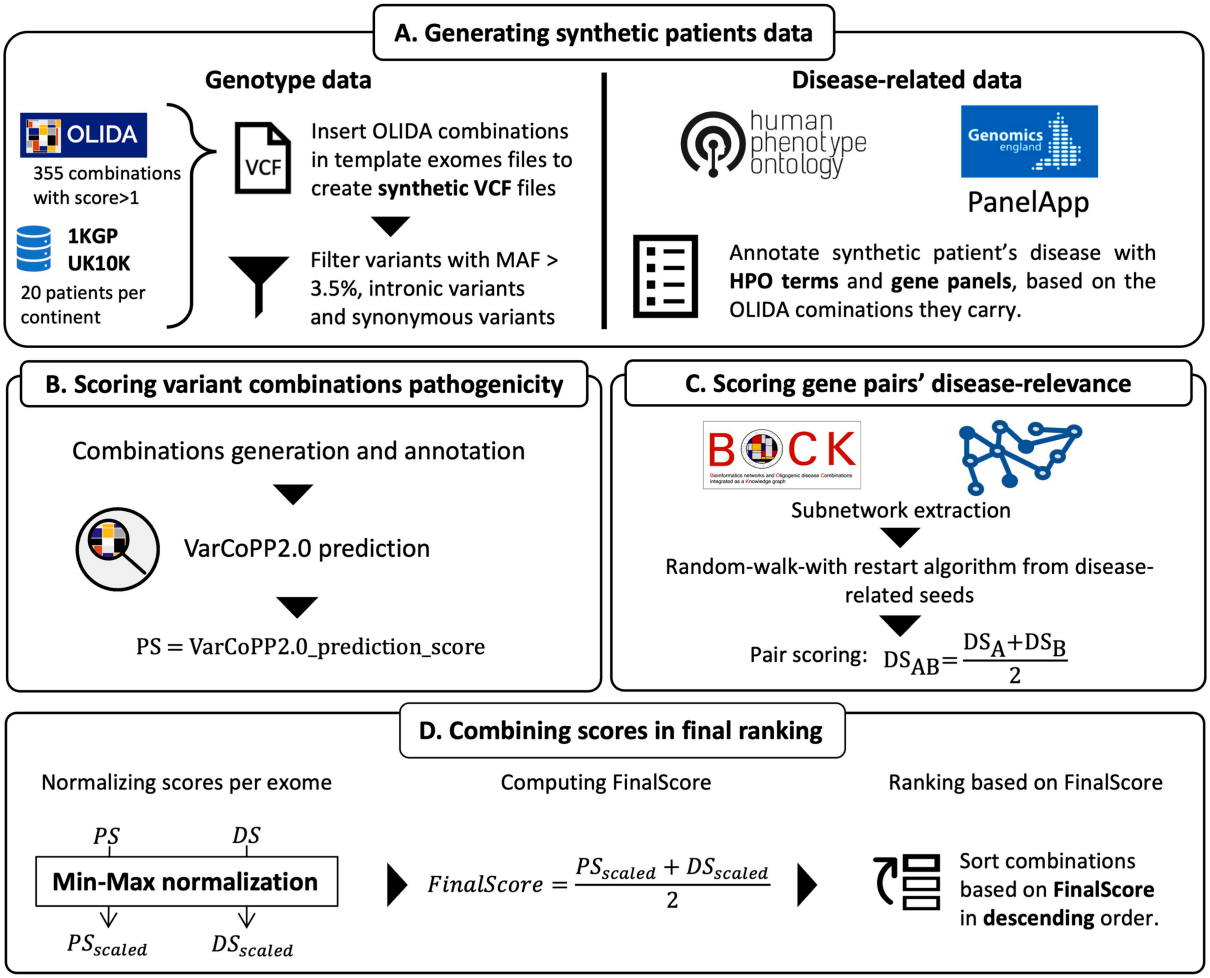
Overview of the developed prioritization method. (A) Synthetic patients’ data are generated by inserting known pathogenic combinations from OLIDA in exomes of the 1KGP and UK10K, filtering the variants based on the VarCoPP2.0 filtering criteria, and annotating them with disease-related phenotypic terms and gene panels, based on the disease associated to the inserted OLIDA combination. (B) All possible variant combinations of variants between two genes are then generated based on the filtered VCF file, and predicted using VarCoPP2.0, resulting in the attribution of a pathogenicity score (*PS*) per variant combination. (C) A disease-relevance score (*DS*) is attributed to all possible gene pairs, by running a random-walk with restart algorithm in BOCK, a heterogeneous network, using the disease-related terms as seeds for the propagation. This algorithm scores the proximity of all genes to the seeds, and the gene-pair score is computed as the average of the two gene scores. (D) The *PS* and *DS* scores are scaled using min–max normalization per exome to have equal weight in the computation of the *FinalScore* (*FS*), which is the average of the aforementioned scores. The combinations are then ranked based on this *FS*, with the highest ranked combination having the highest *FS*.

The *disease relevance score* (*DS*) is obtained by running the RWR algorithm on the heterogeneous graph using as seeds either the HPO terms, the genes belonging to the gene panel, or both types of information. The RWR algorithm outputs a score for nodes of genes, say *A* and *B*, which can be interpreted as a score of proximity between those genes and the set of seeds. In order to obtain the *DS* for a pair of genes *A–B*, the mean of the two individual gene scores (i.e. the mean of *DS_A_* and *DS_B_*) is computed (see Fig. 1C).

### 2.4 Combining the pathogenicity score and disease relevance score for the final ranking

Both *PS* and *DS* are scaled per exome using min–max normalization, such that the combination with the highest pathogenicity has a normalized *PS* (*PS_scaled_*) score of 1 and the combination with the lowest pathogenicity a *PS_scaled_* of 0, and that the combinations with the gene pair with the highest relevance have a normalized *DS* (*DS_scaled_*) of 1, and the combinations with the least relevant gene-pair have a *DS_scaled_* of 0. This normalization ensures equal weight to both types of scores in the computation of the final score. A final ranking score (*FinalScore*) is then computed by taking the average of the *PS_scaled_* and the *DS_scaled_*. The use of other operators for the aggregation of the *PS_scaled_* and the *DS_scaled_* was also investigated and the results are shown in Supplementary Fig. S3. The *FinalScore* (*FS*) ranks all combinations in an exome, with the highest ranking combinations having the highest *FS* (see Fig. 1D).

### 2.5 Comparison with other approaches

The relevance of our novel method is compared in this work to the effectiveness of existing single-variant prioritization methods in finding the digenic variants and to the performance of oligoPVP (Boudellioua *et al.* 2018), which provided an early attempt to rank digenic combinations using a monogenic and PPI perspective. Our aim, with this comparison is 2-fold: We want to show that (i) monogenic approaches to prioritization (whether they are phenotype driven or not) are not sufficient to detect digenic combinations and (ii) that Hop significantly outperforms the only other existing digenic prioritization approach.

CADDv1.6 (GRCh37 assembly) (Rentzsch *et al.* 2018) is used as single-variant method that relies only on genotype data and Exomiserv13.1 (data files v2202 Hg19 assembly run with Java 13.0.2) (Robinson *et al.* 2014) is assessed as it provides a single-variant prioritization method that integrates both genotype and phenotype data. The choice was made to only compare our method with Exomiser as a single-variant phenotype-based prioritizer because it is one of the most widely used methods and a standalone version of the tool can be easily downloaded. The comparison between Hop and monogenic prioritization tools is important, as it is the only way to evaluate the added value of an approach that directly targets oligogenic variants (as it is done in Hop) over single variant approaches. Each method was run using default settings, except for the MAF filter, which was set to match the one of our tool (MAF <3.5%) and with the same filtered “testing” VCFs and the same HPO terms associated to each synthetic patient. For single variant prioritizers, the rank of a combination is measured as the maximum of the ranks of the variants in the combinations, i.e. the minimum number of variants that need to be examined to have all variants from an OLIDA combination in the selection. A variant combination is considered to be in the top 1, if both variants are ranked at the top of the list.

## 3 Results

We introduce here the results obtained with Hop, the proposed oligogenic prioritization method, which for the first time directly uses oligogenic information to effectively prioritize variant combinations in WES data. Hop relies on both pathogenicity information, obtained from VarCoPP2.0 (Versbraegen *et al.* 2023), a digenic variant combination pathogenicity predictor, and information on the patient’s disease, which is propagated in BOCK (Renaux *et al.* 2023), a knowledge graph integrating several biological resources that are relevant to the study of properties and activities of genes and proteins in diseases.

### 3.1 Combining VarCoPP2.0 with phenotype information is essential for efficient prioritization

We first assess how efficient each type of score is for ranking combinations in exomes. To visualize the performance, we plot the Cumulative Density Function curve (CDF), which shows the proportion of exomes for which the inserted oligogenic combination is in the top *K* for varying values of *K*. We present results for *K* ranging up to 50, as we believe this is the maximum number of combinations a user would realistically consider. The results over the whole range of rank values are available in Supplementary Section S7. We compare the ranking performance of using the *PS* alone, i.e. the VarCoPP2.0 predictor, the *DS* alone, i.e. the disease-related information, and the combination of the two scores (*FS*).

**Figure 2.**
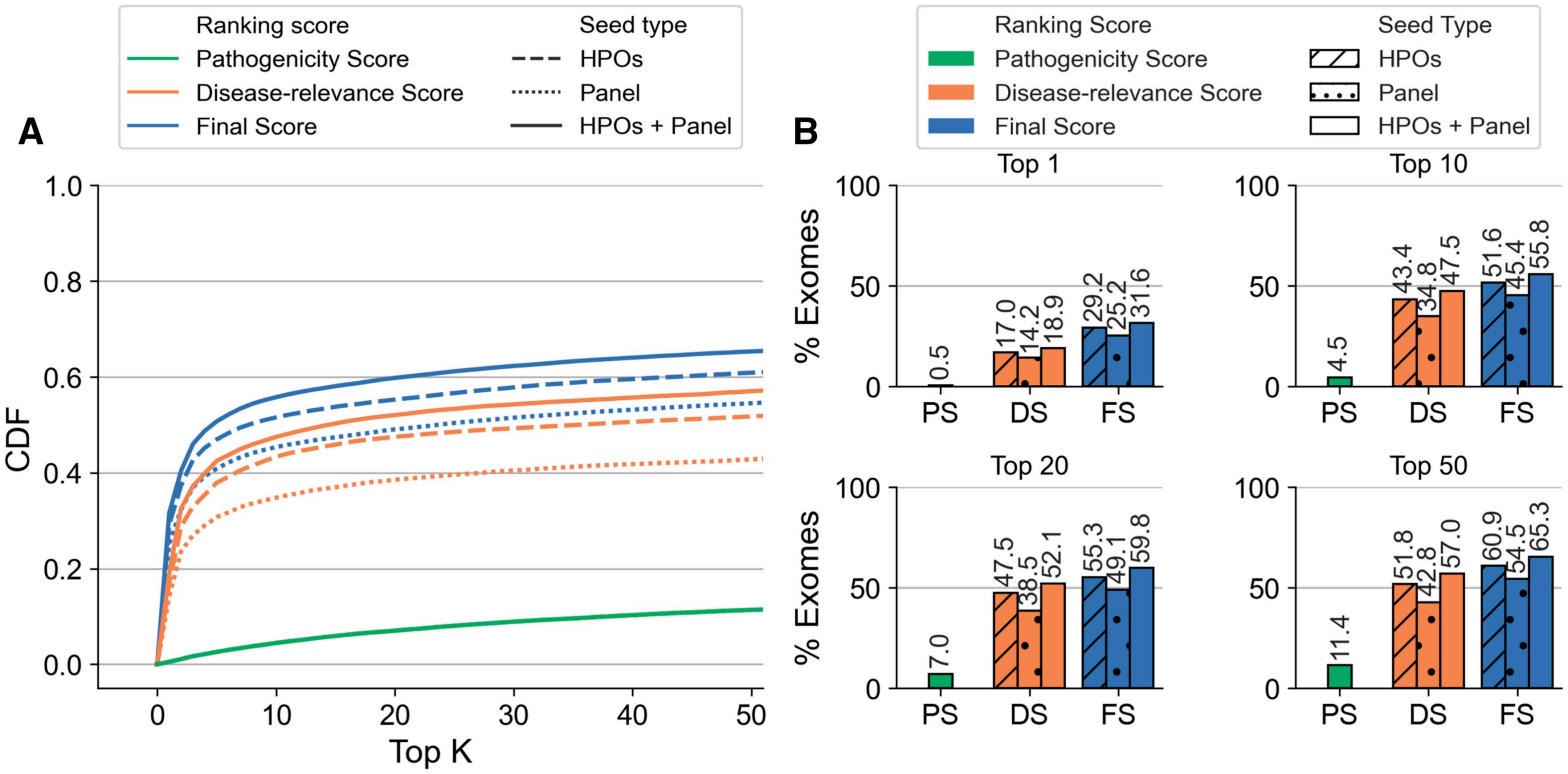
Performance of Hop in the cross-validation exomes. (A) Cumulative Density Function (CDF) plot of the rankings obtained in the cross-validation exomes, by using the *Final Score* (*FS*), *Disease-relevance Score* (*DS*), and *Pathogenicity Score* (*PS*) as ranking scores, with HPO terms as seeds (dashed line), genes from a gene panel as seeds (dotted line) and both HPOs and gene panel as seeds (solid line). The CDF plots illustrate the percentage of exomes for which the OLIDA combination is ranked in the top *K* by each method, with *K* varying between 1 and 50 (inclusive). (B) Percentage of exomes for which the known OLIDA combination is ranked in the top 1, top 10, top 20, and top 50 of the cross-validation exomes based on the different types of scores and seeds for ranking.

**Figure 3.**
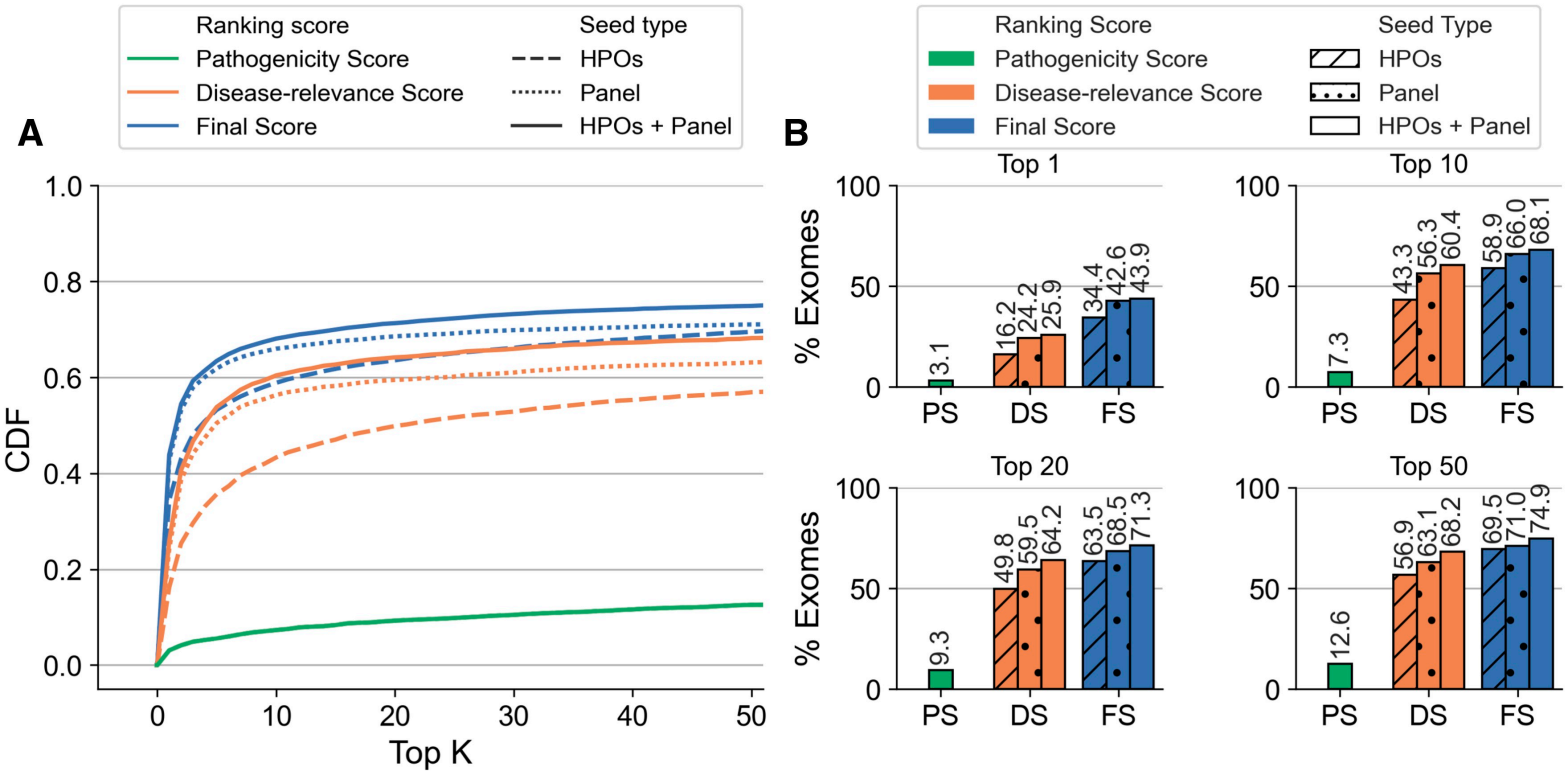
Performance Hop in the independent validation exomes. (A) Cumulative Density Function (CDF) plot of the rankings obtained in the independent validation exomes, by using the *Final Score* (*FS*), *Disease-relevance Score* (*DS*), and *Pathogenicity Score* (*PS*) as ranking scores, with HPO terms as seeds (dashed line), genes from a gene panel as seeds (dotted line) and both HPOs and gene panel as seeds (solid line). The Cumulative Density Function (CDF) plots illustrate the percentage of exomes for which the OLIDA combination is ranked in the top *K* by each method, with *K* varied between 1 and 50 (inclusive). (B) Percentage of exomes for which the known OLIDA combination is ranked in the top 1, top 10, top 20, and top 50 of the independent validation exomes based on the different types of scores and seeds for ranking.

Using, on the one hand, only *PS*, <13% of synthetic exomes had the known pathogenic OLIDA combination in the top 50 of prioritized variant combinations (Figs 2 and 3, green), indicating, as expected, that the VarCoPP2.0 predictor alone is not sufficient for prioritizing digenic combinations in exomes. On the other hand, using only the *DS* based on HPO terms, 52% of the exomes used in cross-validation and 57% of the exomes in the independent set had the OLIDA combination ranked in the top 50 prioritized combinations (Figs 2 and 3, orange). By using *FS*, combining both *PS* and *DS* with HPO terms, 61% of the cross-validation exomes and 70% of the validation exomes contain the known OLIDA combinations in the top 50 (Figs 2 and 3, blue). These results indicate that both *PS* and *DS* must be combined to achieve the highest performance. While different ways of combining these scores were investigated, taking the average of the two scores gives so far the best performance (Supplementary Fig. S3). Overall, in >95% of the exomes, the pathogenic combinations are ranked in the top 1% combinations of the exomes, even though this percentage can represent a large number of combinations for some exomes (the maximum absolute rank of a combination using HOP is 9841606, for OLI694 inserted in an exome containing over 29 million combination, see Supplementary Paragraph S7 of Supplementary Material).

We further investigated the influence of two individual scores, *DS* and *PS*, on the selection of top-ranked combinations (see Supplementary Fig. S4). Our results suggest that the *DS* score provides a slightly stronger contribution to the prioritization of combinations than the *PS* score. Nonetheless, the top-ranked combinations are chosen based on a combination of the scores rather than relying solely on one of the scores alone.

### 3.2 Different sources of disease information improve ranking

We investigate here whether different sources of prior knowledge on the disease can lead to different ranking performances by comparing the use of different seed types for the computation of the *DS* score. HPO terms have been commonly used as prior information in state-of-the-art prioritization methods, providing granularity in the description of a patient’s disease and enabling phenotypic similarity profiling to several diseases, which can lead to the discovery of new gene-disease associations. Notwithstanding their success, other sources of prior information could be useful. Here, we investigate whether gene panels, which are readily used in clinics to assess the possibility of the genetic origin of a disease, provide useful prior information for ranking.

Our results show that using both gene panels and HPO terms as seeds for the prioritization algorithm always leads to an improvement in performance when compared to the use of either type of information alone: In cross-validation, 64.6% of the known pathogenic combinations are identified in the training exomes in the top 50 when both HPO terms and gene panels are used as starting points for the algorithm, as opposed to 60% when only HPO terms are used and 53.6% when only gene panel information is provided (see Fig. 2). In the independent validation, the OLIDA combinations are found in the top 50 combinations in 74.3% of the tested exomes when both types of information are used while this percentage reduces to 68.9% when HPO terms are used alone and of 70.7% when gene panels are used alone (see Fig. 3).

In the cross-validation exomes, using HPO terms as prior-information seems to perform better than using gene panels (Fig. 2), while the converse is true in independent validation exomes (Fig. 3). This difference can partially be explained by the difference in annotation between the combinations in the training set and in the independent validation set. Only 72% of the combinations belonging to the training set could be annotated with a gene panel compared to 87% of the combinations in the test set, which contributed to making this type of information less useful as a seeding strategy in the first case (see Supplementary Table S1). On the other hand, since HPO terms are used to describe patients’ symptoms, all combinations can be annotated with this type of information, which can explain why the performance when using HPO terms appears to be more consistent between the cross-validation and independent exomes.

The performance of Hop appears to be consistent across the different templates used to generate the synthetic exomes. We observe a slightly lower performance when the predictor is used in synthetic exomes generated from individuals of African descent (see Supplementary Fig. S5A). However, the predictor appears to perform equally well when exomes from the UK10K project, which were never included in the training of VarCoPP2.0, are used as templates (see Supplementary Fig. S5B).

### 3.3 Hop prioritizes in a unique manner variant combinations

**Figure 4.**
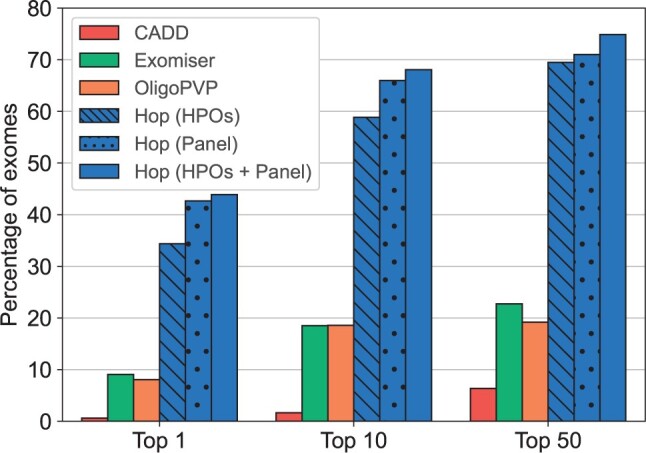
Percentage of exomes for which the OLIDA combinations is ranked in the Top 1, Top 10 and Top 50 instances, when using CADD, Exomiser, OligoPVP, and Hop for prioritization.

In order to evaluate the relevance of digenic prioritization and to compare the performance of our method to state-of-the-art tools, we assessed the performance of three alternative prioritization methods on our independent validation dataset: (i) CADD (v1.6), which is a single variant prioritizer using only genotype information, (ii) Exomiser (v13.1), which is a single-variant prioritizer using both phenotype and genotype information, and (iii) OligoPVP, which integrates monogenic prioritization together with PPI information, making it a first attempt to oligogenic phenotype-based prioritization. Whereas (i) and (ii) serve to show the added value of prioritization of variant combinations directly, (iii) is used to compare the performance. These comparisons are made against the different versions of our tool (i.e. using HPO terms, gene panel and both types of information), with the results shown in Fig. 4.

The results show that Hop, even using only HPO terms as seeds, is more adequate for identifying the (potential) digenic origins of a disease as it ranks the relevant variants earlier (and in combination) than monogenic prioritizers: Hop ranks the known pathogenic combination in the top 50 in 69.5% of the synthetic exomes, while CADD and Exomiser only identified the relevant variants involved in the combination in the top 50 in 6.4% and 22.7% of the exomes respectively. OligoPVP, the only attempt so far to perform digenic ranking, only identified the variants involved in the combination in the top 50 in 19.2% of the exomes, demonstrating the usefulness of our Hop approach for digenic prioritization.

## 4 Discussion and conclusion

We present Hop, an original and relevant prioritization method that directly uses oligogenic information at several biological levels in order to rank, in WES data, variant combinations based on how likely they are to explain a patient’s phenotype. Hop integrates a pathogenicity score (*PS*) together with a disease-relevance score (*DS*), in order to prioritize the combinations. The *DS* is computed by propagating information about the patient’s disease in a heterogeneous network, in order to score the proximity of each gene pair to a set of user-defined seeds.

With WES data becoming more easily accessible, the development of oligogenic prioritization is becoming increasingly important as current oligogenic prediction methods were not designed for analyzing such quantities of data. Indeed, although the VarCoPP2.0 predictor used in this work to obtain the pathogenicity score was shown to have high accuracy in a classification setting (Versbraegen *et al.* 2023), as a prioritization method, it only ranked the known pathogenic combination in the top 50 in <13% of the exomes. This implies that a clinician wanting to identify potentially pathogenic variant combinations in a patient’s WES data would need to manually curate a huge number of combinations and investigate their disease relevance in order to identify the potentially disease-causing combination. With Hop, the disease-relevance of gene pairs is scored and combined with the pathogenicity prediction in order to provide the clinician with a ranking of the combinations. We show that by only looking at the top 10 combinations of this ranking, known disease-causing variant combinations can be found in 59.3% of all synthetic exomes (cross-validation and independent set). This significantly reduces the amount of work of the clinician, since, in order to obtain the same accuracy using VarCoPP2.0 alone, >3000 variant combinations would need to be investigated.

Moreover, Hop is shown to outperform existing monogenic approaches on the task of ranking digenic variant combinations in WES data. This does not diminish the usefulness of existing monogenic approaches, but rather highlights not only the need to design tools specifically for this task, but also the importance of scoring pathogenicity of variant combinations using method trained on oligogenic disease cases. Indeed, variants involved in oligogenic combinations have been shown to have different characteristics than variants involved in monogenic diseases, such as higher MAF and lower monogenic pathogenicity scores for both digenic and modifier variants (Gazzo *et al.* 2017, Versbraegen *et al.* 2019, Papadimitriou *et al.* 2023). Hop also introduces a new method to score the disease-relevance of gene pairs, combining the scores of the RWR algorithm to obtain a gene-pair proximity score to a set of disease-related seeds. Although prioritization methods have traditionally relied on phenotype-based approaches, we further investigate the utilization of gene panels as an alternative source of information for disease-relevance scoring. We demonstrate that incorporating both phenotypic and panel data can significantly enhance performance. Moreover, by accommodating both types of information, Hop can perform prioritization even in cases where the patient’s phenotype is unknown, as long as a gene panel for the disease is available.

Although Hop is highly successful, the predictor’s performance appears to plateau, even when both HPO terms and disease genes are given as prior information. The first possible explanation for this limitation is that Hop strongly relies on prior knowledge and is thus limited by the incompleteness of the available molecular knowledge. This is especially true when such knowledge is obtained through low throughput acquisition methods, such as the PPI or gene ontology networks used in this work (Menche *et al.* 2015). In addition to the limitations caused by incompleteness, using prior knowledge can introduce important biases. This can be seen, e.g. by the fact that digenic genes and disease genes in general are more connected in any knowledge graph than human genes overall (see Supplementary Fig. S2 in case of BOCK). In the case of oligogenic disease, it is possible that the bias present in the HPO to gene network, where only 23.5% of all human genes are linked to HPO terms, introduces limitations. Indeed, the link between an HPO term and a gene is created based on known gene-to-disease associations, from OMIM or Orphanet, and disease-to-HPO associations, from the HPO database (Köhler *et al.* 2014). However, these databases contain almost exclusively data on monogenic diseases, which implies that genes involved in oligogenic diseases have fewer HPO terms associations than other disease genes, and is thus likely to explain why knowledge-based approaches for oligogenic diseases are currently limited by prior knowledge.

The limitations introduced by knowledge bias is further illustrated in our results by the difference in performance between the cross-validation exomes and the exomes of the independent set. Indeed, more combinations in the independent set could be annotated with gene panels, indicating that these combinations were linked with diseases that are overall more studied. This is likely to explain why our knowledge-based predictor also predicts these combinations better, especially knowing that, for 79% of the combinations that have an associated panel, the two genes involved in the combinations are present in the gene panel.

Furthermore, it should be emphasized that Hop, like any prioritization method, provides a ranked list of variant combinations for all exomes, without offering confidence estimates or suggesting how many candidates should be kept for further investigation. Given the uncertain frequency of digenic inheritance in diseases, this is important to consider when using the tool. Hop is most effectively used as a complementary approach to existing methods or in case-control studies for diseases with suspected digenic inheritance. For example, it can be applied to patients for whom a monogenic diagnosis could not be obtained using traditional approaches, or to detect genetic modifiers to these monogenic variants.

Finally, it is important to note that the performance of Hop has so far only been evaluated using synthetic data, and it will be very important to assess the performance of the tool on real clinical WES data. It will also be essential to assess whether using such a prioritization approach can be helpful when analyzing not only single patients but also patient cohorts and assess whether such an approach can help identify oligogenic signatures for specific diseases. The next important step is therefore to make the method available for exhaustive testing by researchers and clinicians while ensuring privacy preservation of patients’ data. This could be done by integrating the tool into the ORVAL platform (Renaux *et al.* 2019), providing the tool in a cloud infrastructure or making it available through the Galaxy platform (Afgan *et al.* 2016).

## Supplementary Material

btae184_Supplementary_Data

## Data Availability

The BOCK knowledge graph is available in Zenodo (doi: 10.5281/zenodo.7185679). The annotated data for the 1KGP individuals and the OLIDA combinations is available in Zenodo (doi: 10.5281/zenodo.7185679). The code is available at https://github.com/oligogenic/HOP.
