# Supplementary material for: Prioritization of oligogenic variant combinations in whole exomes
Source: Bioinformatics. 2024 Apr 11;40(4):btae184. doi: 10.1093/bioinformatics/btae184 (PMC11037482; doi:10.1093/bioinformatics/btae184)
Supplement: btae184_Supplementary_Data [file btae184_supplementary_data.zip › Supplementary_file.pdf]

Prioritization of oligogenic variant combinations in whole exomes - Supplementary Information

Barbara Gravel, Alexandre Renaux, Sofia Papadimitriou, Guillaume Smits, Ann Nowé and Tom Lenaerts

S1 The VarCoPP2.0 predictor

For pathogenicity scoring, Hop uses VarCoPP2.0, a variant combination pathogenicity predictor [21]. This predictor is a machine learning model that has been trained on high-quality instances from OLIDA as positive instances and combinations from individuals of the 1KGP as negative instances. The positive combinations were selected from OLIDAv1 based on their confidence scores, in order to construct a dataset with combinations associated with at least a weak level of confidence. Only combinations with a FINALmeta score of 1 and above, and which involved variants in two genes were included. Variants for the 1KGP individuals were filtered to have similar characteristics to the positive set variants: variants with MAF $> 3.5\%$ were removed as well as intronic variants and synonymous variants which were further than 195 nucleotides from exon boundaries. These criteria are based on the highest MAF and the largest distance from exon boundaries found for variants involved in training combinations from the OLIDA database.

Instances in the training are annotated with 15 features from the different biological levels. This feature set was determined based on manual literature search for relevant metrics, as well as a feature reduction algorithm that identified the most relevant features for the classification problem at hand. At the variant level, the CADD raw score [17] is used as a measure of deleteriousness. Variants were scored using CADDv1.6 for GRCh37 assembly. At the gene level, combinations were annotated with a measure of haploinsufficiency from HIPred [18], the dN/dS ratio as an evolutionary feature and the Inheritance Specific Pathogenicity Predictor (ISPP) [8] as a measure of gene pathogenicity. At the gene pair level, combinations were annotated with the Biological Distance, a measure of the distance between the genes in a PPI network, a Biological Process Gene Ontology similarity score, and a KG distance measure, which measures the distance between the two genes in the BOCK knowledge graph. More details on how these features were computed can be found in the original publication [21]. The final model is a balanced random forest classifier, implemented using the Imbalanced-learning python package [12].

In this work, since the OLIDA combinations that were inserted to generate synthetic exomes overlap with the training set of the VarCoPP2.0 predictor, we therefore implemented the pathogenicity scoring in two different ways: (1) For exomes in which the combination inserted was part of the training set of VarCoPP2.0, the pathogenicity score was obtained using a VarCoPP2.0 model retrained in a cutom cross-validation procedure (see paragraph below), (2) for exomes in which the combination inserted was not part of the training set of VarCoPP2.0, the final model trained on the full training set is used.

All OLIDA combinations of sufficient quality were annotated with the relevant features using an in-house database (which contains all up to date feature measures, see Table 1 of [21]), producing a training set. For the“training” exomes, the training set was divided in 10 folds, ensuring that combinations for which the variants were located in the same gene pair belonged to the same fold. For each fold (referred to as test fold), a model with the same structure as the VarCoPP2.0 model was retrained on the remaining 9 folds, and used to predict the combinations of the exomes in which the combinations from the test fold were inserted. This procedure ensures that the VarCoPP2.0 model used to score the pathogenicity of the combinations has never seen the combinations that it is predicting. For the testing exomes, the final VarCoPP2.0 model, trained on the full training set, was used to assign a pathogenicity score. These testing exomes consisted of the 1KGP and UK10K exomes in which we inserted OLIDA combinations of sufficient quality (FINALmeta > 0) which were included in OLIDA versions 2 and 3 and were thus not present in the training set of VarCoPP2.0.

S2 Bioinformatics networks and Oligogenic Combinations as a Knowledge graph (BOCK)

Hop uses as background information a knowledge graph specifically designed for the study of genetic interactions in oligogenic diseases, which schema is shown below. This knowledge graph integrates information from 12 different biological databases, together with oligogenic information from the OLIDA database. The biological databases were selected based on their public accessibility, their relevance for studying human diseases, and their clear curation policies. In addition, included resources cover a minimum of 20% of all human genes at different biological levels.

Integrated biological information include: Protein-protein interactions (PPI), collected from the Mentha database [6], coexpression between genes, obtained from post-processed GTEx data collected in the TCSBN database [11], sequence similarity data between genes, downloaded from STRING [19], molecular functions, biological processes, cellular components and their linked genes, collected from the Gene Ontology [3, 1], protein domain and family information, collected from the InterPro database [5], protein complexes information, gathered from CORUM [7] and phenotype and disease information collected from the Human Phenotype Ontology [10].

The full knowledge graph is available at https://doi.org/10.5281/zenodo.7185680 and more details about is construction is available at https://doi.org/10.1186/s12859-023-05451-5. Additional details on the KG construction are provided in Supplementary Table A1 and A2 of the aforementioned article.

In order to compute the disease-relevance score, the knowledge graph was filtered to remove the “Disease” and “Oligogenic Combination” nodes as well as the edges connecting to these nodes (“involves”, “causes” and “described” edges, see Figure S1). This filtering resulted in a heterogeneous network with 8 layers: Gene, Phenotype, Protein Domain, Protein Family, Protein Complex, Molecular Function, Biological Process and Cellular Component.

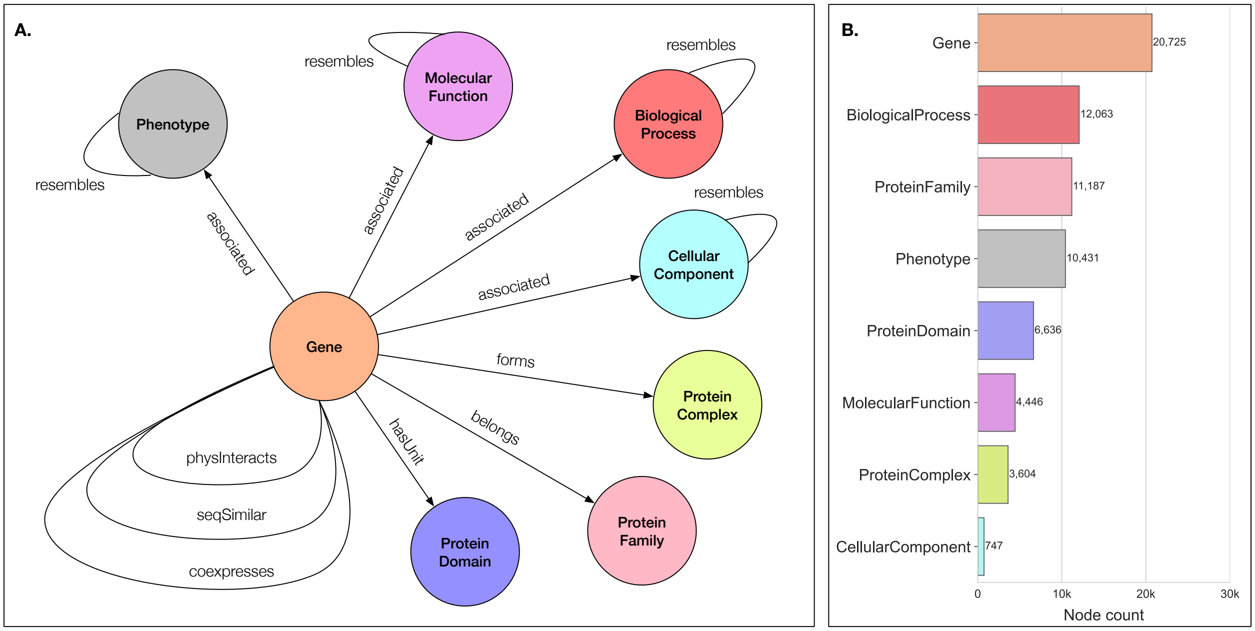

Figure S1: Relevant part of the BOCK knowledge graph used in Hop. Compared to the original BOCK [16], Disease and OligogenicCombination nodes have been filtered. (A) Schematic representation of the knowledge graph with the different entities represented as nodes of different colors and the different types of edges represented as labeled arrows. (B) Node counts for each of the biological entities present in the graph. Note that only nodes with at least one neighbor are counted, as disconnected nodes are irrelevant in Hop. Figure adapted from [16]

S3 Analysis of gene set degrees in BOCK and knowledge induced biases

In order to better quantify the potential biases implied by using a knowledge-graph approach, we here assess whether certain sets of genes are more likely to be found by the random walk algorithm. To do this, we investigate **the degree of a node**, which assesses the number of nodes a node is connected to. We computed this value for each node in the network and then visualize and quantify the difference in distribution between 4 sets of genes:

1. **Digenic genes used in training (N=323):** All genes that are known to be involved in a digenic disease with sufficient confidence, and which have been used in the training set of Hop.

2. **Oligogenic genes (N=762):** All genes that are found to be associated with an oligogenic disease in OLIDAv2.

3. **Disease genes (N=4487):** All genes which are associated with a disease in the Orphanet database.

4. **All human genes (N=20725):** All human genes which are connected in the graph.

The distributions of degrees are shown in Figure S2. In order to test for significance in the difference between the distributions, we used the Kolgomorov-Smirnov statistical test [14], and used Bonferoni correc-

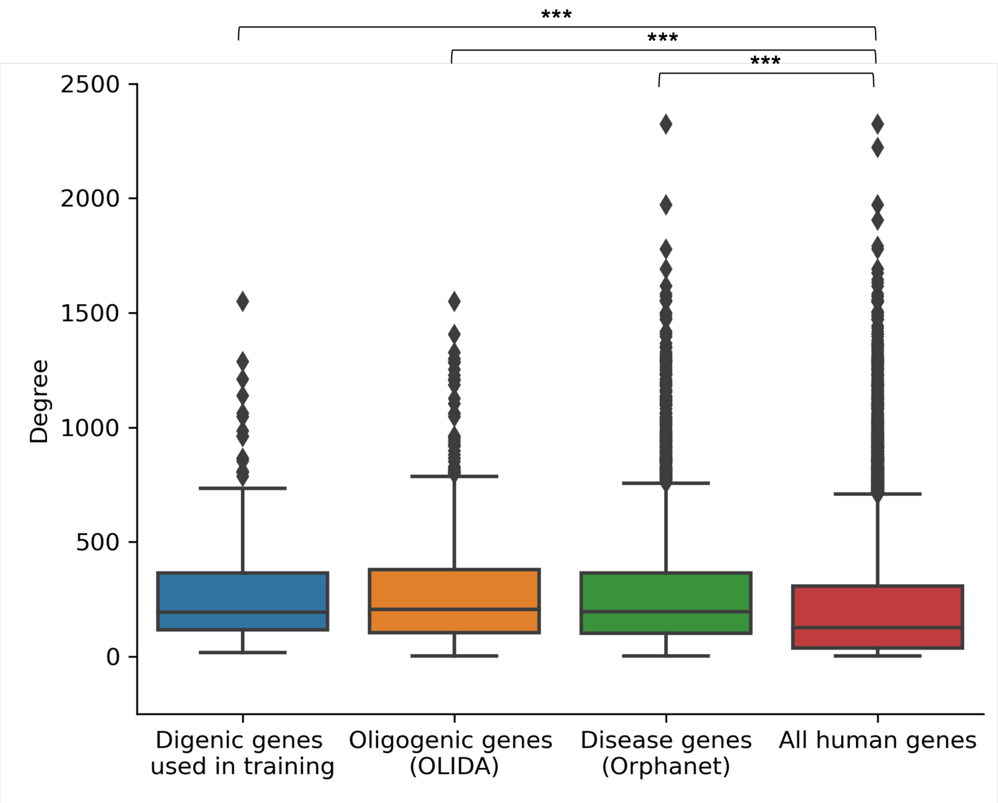

Figure S2: Distribution of the degrees of different gene sets in BOCK.

tion [2] to adjust the p-values for the 6 pairwise comparisons. Only comparisons with p-values < 0.05 are shown in Figure S2.

We observe that gene sets containing genes invoved in disease (oligogenic or monogenic, blue, orange and green boxplots in the Figure S2) all show significant difference with all human genes (Red boxplot), and that they typically present with a higher median degree. However, the distribution of degrees in oligogenic genes does not differ significantly from the distribution of degrees in monogenic genes. This highlights the fact that the bias present in BOCK is probably due to study bias, and it is not specific for oligogenic genes.

S4 Parameter analysis

Except for the parameters of the VarCoPP2.0 model, the described method relies on only one parameter, which controls the spread of the random-walk-with-restart algorithm. We therefore evaluated the influence of this parameter on our results by reproducing our results in a subset of the synthetic exomes (using 50 of the 1KGP exome templates) with the restart probability varying between 0.1 and 0.9 with intervals of 0.2.

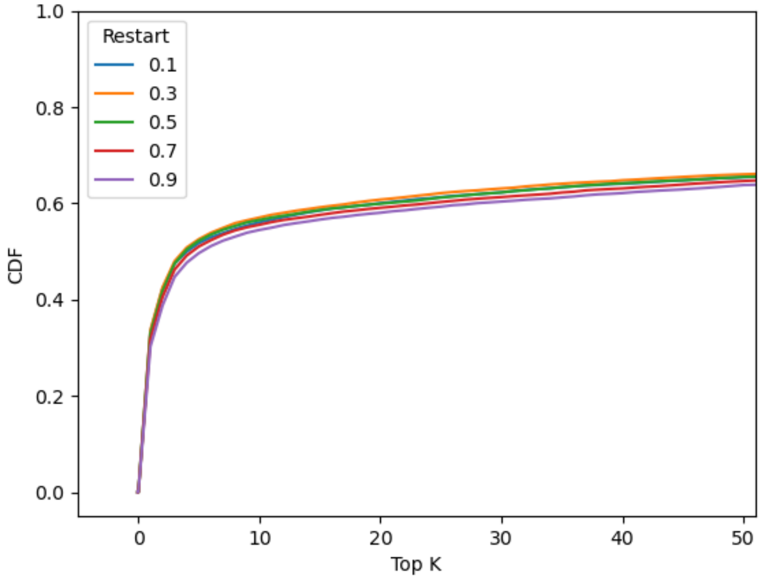

Figure S3: Cumulative density function plot of the rankings obtained in the cross-validation exomes, with the HPO and gene panel seeds for different values of the restart parameter for the computation of the DS. The CDF plots illustrate the proportion of exomes for which the OLIDA combination is ranked in the top k for k varied between 1 and 50 (inclusive).

The results, presented in Figure S3, show that this parameter has very little effect on the percentage of exomes for which the known OLIDA combination is ranked in the top K when K is lower than 50. This is consistent with the conclusions of other works on RWR algorithms in biological networks, which have shown that except for extreme values, this parameter did not have a strong effect on the output ranking [20, 9, 13].

Based on these results, we decided to set the restart to 0.3 for the rest of the analyses.

S5 Effect of different operators for the combination of the disease-relevance score and the pathogenicity score

Different operators were tested to combine the disease relevance score and the pathogenicity score in a final ranking score. We evaluated using the minimum of the two scores, the maximum of the two scores, the average of the two scores and the multiplication of the scores.

Except for the maximum of the two scores, all other operators appeared to perform similarly well. The average of the PS and the DS seemed to slightly outperform the other operators and was thus used to compute the $FinalScore$.

The fact that the maximum of the two scores did not perform as well as is probably due to the fact that this operator does not put any minimum condition on each of the two scores. This leads to any combination with any one of the two scores as high to be positioned highly in the ranking (even combinations that are predicted as highly pathogenic but in genes that are not relevant to the disease of interest or vice-versa) and therefore will not put emphasis on ranking highly the combinations that are both "pathogenic" and relevant to the disease.

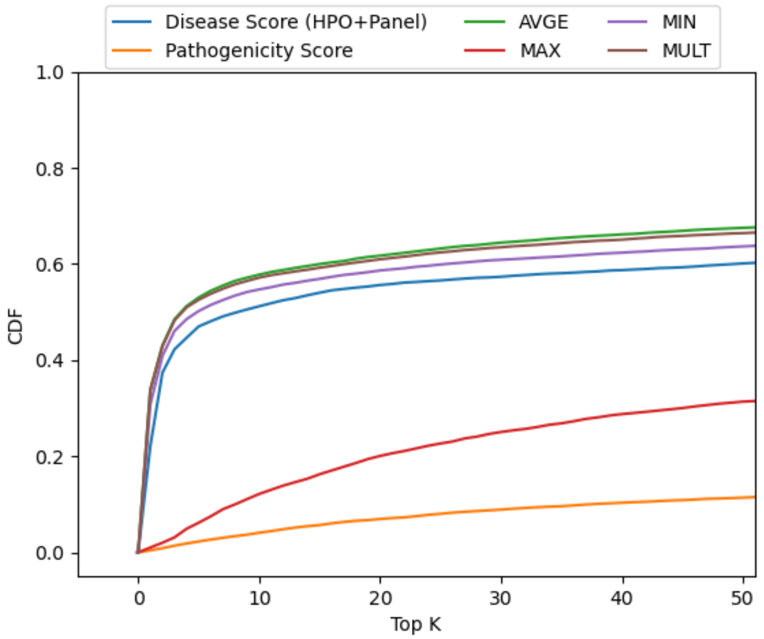

Figure S4: Cumulative density function plot of the rankings obtained in the cross-validation exomes, with the HPO and gene panel seeds for the pathogenicity and disease scores and for the four different operators tested to combine the two scores. The CDF plots illustrate the proportion of exomes for which the OLIDA combination is ranked in the top k for k varied between 1 and 50 (inclusive).

S6 Analysis of the relative contribution of each score to the final ranking

In order to better understand how the final ranking is created, we decided to investigate how the two scores contribute to the final ranking in order to see whether one of the scores has (or not) a stronger effect than the other.

In order to do this, we decided to focus on the combinations that are ranked at the top, since these are the combinations that are considered to be the most relevant and which will require further investigation by the clinician. The objective is to determine whether one of the individual scores has a stronger impact on the selection of the top-ranked combinations.

We therefore measure, for each synthetic exome used in cross-validation, how many combinations present in the top K based on the FS were also in the top K based on both PS and DS scores individually and together. This is done for the rankings using both HPO terms and gene panels as prior information since these seeds lead to the highest performance.

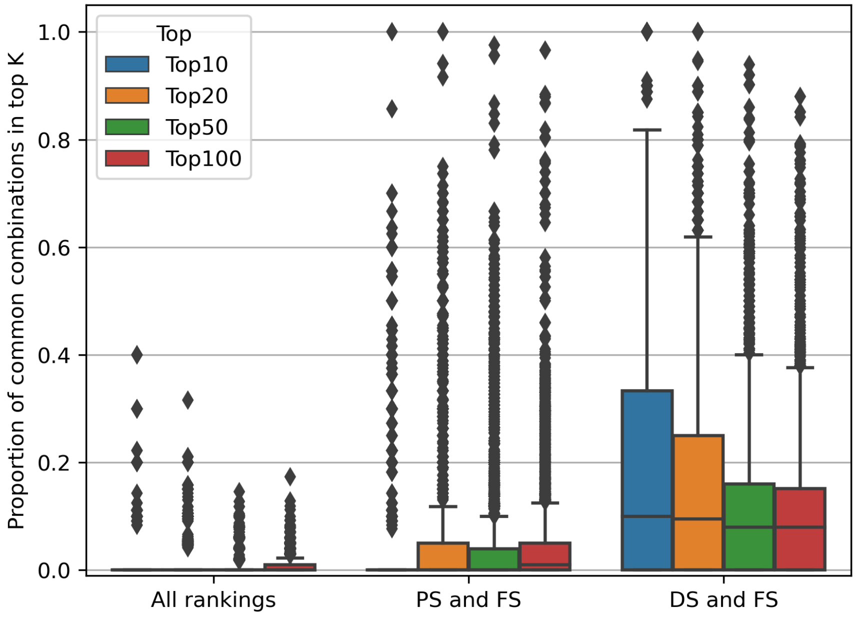

Figure S5: Proportion of common combinations at the top K of the rankings generated by the different scores for K values of (10, 20, 50 and 100). All rankings represent the proportion of the combinations in the top K of the ranking based on the FS that are also in the top K of the combinations based on the PS and in the top K of the combinations based on the DS. PS and FS represent the proportion of the combinations in the top K of the ranking based on the FS that are also in the top K of the combinations based on the PS. PS and FS represent the proportion of the combinations in the top K of the ranking based on the FS that are also in the top K of the combinations based on the PS. Each data point comes from one of the cross-validation synthetic exomes. In this analysis, both HPOs and Panel were used as seeds.

The results, presented in Figure S5, indicate that very few of the combinations with the highest FS are

consistently in the top K generated by both individual scores. Comparing the overlap between the highly prioritized combinations based on the FS and the DS shows that a small proportion of combinations is often found in common, and that this proportion decreases as we increase the size of the top combinations considered. This overlap is larger compared to the overlap between the top combinations based on the FS and the top combinations based on the PS. This suggests that the DS score provides a slightly stronger contribution to the highly ranked combinations compared to the PS score.

However, despite this observation, the number of overlapping combinations between any individual score and the FS remains relatively low (the median of the percentage of common top combinations between the DS and $FinalScore$ is less than 20%). This implies that the top-ranked combinations were primarily selected based on a combination of the scores rather than relying solely on one of the scores alone.

S7 Analysis of the performance of Hop across the whole range of ranks

The results in the main manuscript mostly show the performance of the predictor for combinations which are ranked between the 1st and 50th absolute ranks. In this section, we present the performance of the tool over the full range of possible ranks. The results are shown in Figure S6, for the cross-validation and independent exomes combined. We decided to show both the results in terms of absolute rank (S6A) and in terms of percentile rank (S6B), since it is important to highlight that the exomes could contain a wide range of numbers of combinations that needed to be prioritized.

The plot shows that for the large majority of the exomes (over 99%), the known pathogenic combinations were ranked in the top 6% of the exome, although this can represent up to 372000 in absolute rank, due to some exomes containing a very large number of combinations.

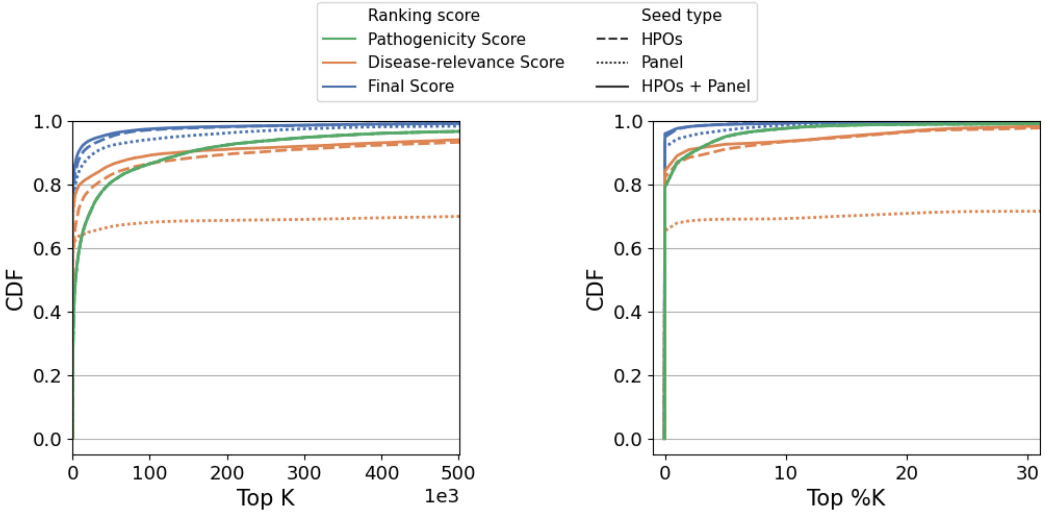

Figure S6: Performance of Hop in the cross-validation and independent exomes over a large range of ranks. (A) Cumulative Density Function (CDF) plot of the rankings, by using the FS (blue), DS (Orange), and PS (green) as ranking scores, with HPO terms as seeds (dashed line), genes from a gene panel as seeds (dotted line) and both HPOs and gene panel as seeds (solid line). The CDF plots illustrate the percentage of exomes for which the OLIDA combination is ranked in the top K by each method, with K varying between 1 and $500x1e^{-3}$ (inclusive). (B) Cumulative Density Function (CDF) plot of the rankings, by using the FS (blue), DS (Orange), and PS (green) as ranking scores, with HPO terms as seeds (dashed line), genes from a gene panel as seeds (dotted line) and both HPOs and gene panel as seeds (solid line). The CDF plots illustrate the percentage of exomes for which the OLIDA combination is ranked in the top K percentile by each method, with K varying between 1 and 30% (inclusive).

S8 Analysis of the performance of the tool per continent

For our synthetic patients, we used as exome template the VCF files of individuals from the 1000 genomes project originating from different continents (we selected 20 individuals per continent) as well as 20 individuals from the UK10K ALSPAC cohort. This allows us to analyse whether Hop performs differently based on the ethnicity of the synthetic patient. The results presented in Figure S7 shows that in both cross-validation exomes and independent exomes the tool seem to perform less well in exomes generated with VCF files of individuals from African descent. This can be mostly explained by the fact that individuals from African origin typically present with a larger number of genetic variants.

The performance of the tool is similar and even slightly better in the exomes that were generated by using data from the UK10K project as templates. This shows that the performance is not biased by the fact that part of the training set of VarCoPP2.0 was obtained from individuals of the 1KGP.

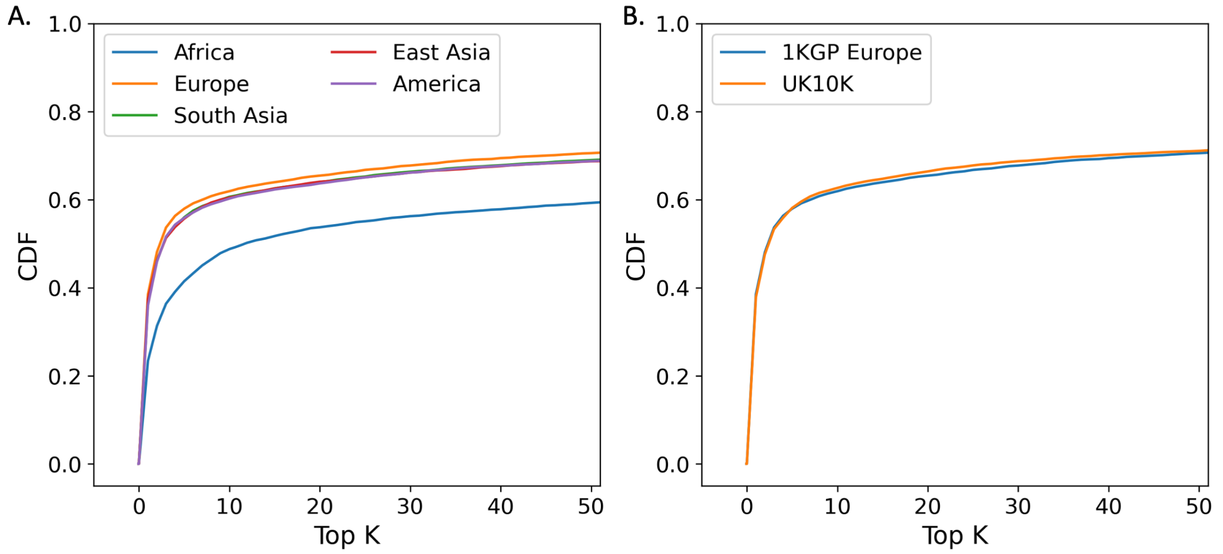

Figure S7: Cumulative density function plot of the rankings obtained by using the FS in all synthetic exomes (cross-validation and independent) comparing based on the ethnicity of the exome template in the 1KGP (A) and comparing based on the dataset used (1KGP or UK10K) for Europeans (B). Each line represent the rankings of the 420 OLIDA combination inserted in 20 different exome templates. The CDF plots illustrate the percentage of exomes for which the OLIDA combination is ranked in the top k by each method, with k varied between 1 and 50 (inclusive).

S9 Statistics on the differential annotation between the cross-validation synthetic patients and the independent set synthetic patients

The generated synthetic patients were separated in two groups: "training" synthetic patients, for which the inserted OLIDA combinations belonged to the training set of the VarCoPP2.0 predictor, and "testing" synthetic patients for which the inserted OLIDA combination was independent from the training set of the VarCoPP2.0 predictor. These synthetic patients were then annotated with HPO terms and gene panel to describe the disease associated with the OLIDA combination. Since not all diseases have an associated gene panel, this resulted in a differential annotation between the "training" synthetic patients and "testing" synthetic patients which led to slight differences in the performance of the tool when it was evaluated in cross-validation or in independent validation. This difference in annotation between these two sets is quantified in the following table.

	HPO terms	Gene Panel	HPO + Panel
	Cross-validation exomes		
Percentage of annotated combinations	100%	72%	100%
Average number of seeds per combination	4.80 ($\sigma = 3.81$)	112.29 ($\sigma = 185.73$)	116.17 ($\sigma = 184.93$
	Independent validation exomes		
Percentage of annotated combinations	100%	87%	100%
Average number of seeds per combination	4.14 ($\sigma = 3.15$)	66.70 ($\sigma = 141.26$)	70.84 ($\sigma = 140.67$)

Table S1: Percentage of combinations that are annotated by HPO terms and gene panels and average number of terms/genes associated with each combination for the three types of seeds investigated.

**Hop**	Version	1.0
**Data**	Provenance	OLIDA [15], 1000 genomes Project [4] and UK10K project
	Dataset splits	301 positive instances for training data and 119 positive instances for validation set. 120 neutral individual exomes (20 from each continent of the 1KGP project and 20 from the UK10K project). Training with a custom 10 fold cross-validation stratified by gene pair.
	Redundancy between data splits	Templates for the synthetic exomes are the same between the cross-validation and the independent exomes
	Availability of data	yes: olida.ibsquare.be (new curated data will be added) and www.internationalgenome.org. For UK10K data, access must be asked to the DAC of the project
**Optimization**	Algorithm	Balanced Random Forest for VarCoPP2.0 and RWR for the disease-relevance score.
	Meta-predictions	yes: CADD features and ISPP features stem from a predictive model.
	Data encoding	global features
	Parameters	400 decision trees within RF and 0.3 restart parameter for the RWR algorithm
	Features	15 features of the VarCoPP2.0 model and scores of the RWR algorithm
	Fitting	decision trees of the VarCoPP2.0 model are pruned to avoid overfitting
	Regularization	No
	Availability of configuration	yes, github.com/oligogenic/Hop
**Model**	Interpretability	Feature importances can be used for VarCoPP2.0 since it is a random forest model. Contribution of the disease-relevance and the pathogenicity scores can be dissected.
	Output	Combinations scored with 3 types of scores: Pathogenicity, Disease-relevance and FinalScore and a ranking of the top N combinations based on the FinalScore.
	Execution time	1 synthetic exome in 431s
	Availability of software	Github: github.com/oligogenic/Hop
**Evaluation**	Evaluation method	Both stratified cross-validation and independent validation data
	Performance measures	Cumulative density function and percentage of exomes with the combination in the top K.
	Comparison	Comparison with existing monogenic prioritizer and oligoPVP, a first attempt at digenic prioritization
	Confidence	performance differences apparent
	Availability of evaluation	yes, Github: github.com/oligogenic/Hop

Table S2: DOME Table consisting of essential information to assess the machine learning approach [22].

References

[1] The Gene Ontology resource: enriching a GOld mine. *Nucleic acids research*, 49(D1):D325–D334, 1 2021.

[2] R. A. Armstrong. When to use the Bonferroni correction. *Ophthalmic & physiological optics : the journal of the British College of Ophthalmic Opticians (Optometrists)*, 34(5):502–508, 9 2014.

[3] M. Ashburner, C. A. Ball, J. A. Blake, D. Botstein, H. Butler, J. M. Cherry, A. P. Davis, K. Dolinski, S. S. Dwight, J. T. Eppig, M. A. Harris, D. P. Hill, L. Issel-Tarver, A. Kasarskis, S. Lewis, J. C. Matese, J. E. Richardson, M. Ringwald, G. M. Rubin, and G. Sherlock. Gene ontology: tool for the unification of biology. The Gene Ontology Consortium. *Nature genetics*, 25(1):25–29, 5 2000.

[4] A. Auton, L. D. Brooks, R. M. Durbin, E. P. Garrison, H. M. Kang, J. O. Korbel, J. L. Marchini, S. McCarthy, G. A. McVean, and G. R. Abecasis. A global reference for human genetic variation. *Nature*, 526(7571):68–74, 10 2015.

[5] M. Blum, H.-Y. Chang, S. Chuguransky, T. Grego, S. Kandasaamy, A. Mitchell, G. Nuka, T. Paysan-Lafosse, M. Qureshi, S. Raj, L. Richardson, G. A. Salazar, L. Williams, P. Bork, A. Bridge, J. Gough, D. H. Haft, I. Letunic, A. Marchler-Bauer, H. Mi, D. A. Natale, M. Necci, C. A. Orengo, A. P. Pandurangan, C. Rivoire, C. J. A. Sigrist, I. Sillitoe, N. Thanki, P. D. Thomas, S. C. E. Tosatto, C. H. Wu, A. Bateman, and R. D. Finn. The InterPro protein families and domains database: 20 years on. *Nucleic acids research*, 49(D1):D344—D354, 1 2021.

[6] A. Calderone, L. Castagnoli, and G. Cesareni. mentha: a resource for browsing integrated protein-interaction networks. *Nature Methods*, 10(8):690–691, 2013.

[7] M. Giurgiu, J. Reinhard, B. Brauner, I. Dunger-Kaltenbach, G. Fobo, G. Frishman, C. Montrone, and A. Ruepp. CORUM: the comprehensive resource of mammalian protein complexes-2019. *Nucleic acids research*, 47(D1):D559–D563, 1 2019.

[8] J. S. Hsu, J. S. Kwan, Z. Pan, M. M. Garcia-Barcelo, P. C. Sham, and M. Li. Inheritance-mode specific pathogenicity prioritization (ISPP) for human protein coding genes. *Bioinformatics*, 32(20):3065–3071, 10 2016.

[9] S. Köhler, S. Bauer, D. Horn, and P. N. Robinson. Walking the Interactome for Prioritization of Candidate Disease Genes. *The American Journal of Human Genetics*, 82(4):949–958, 4 2008.

[10] S. Köhler, M. Gargano, N. Matentzoglu, L. C. Carmody, D. Lewis-Smith, N. A. Vasilevsky, D. Danis, G. Balagura, G. Baynam, A. M. Brower, T. J. Callahan, C. G. Chute, J. L. Est, P. D. Galer, S. Ganesan, M. Griese, M. Haimel, J. Pazmandi, M. Hanauer, N. L. Harris, M. J. Hartnett, M. Hastreiter, F. Hauck, Y. He, T. Jeske, H. Kearney, G. Kindle, C. Klein, K. Knoflach, R. Krause, D. Lagorce, J. A. McMurry, J. A. Miller, M. C. Munoz-Torres, R. L. Peters, C. K. Rapp, A. M. Rath, S. A. Rind, A. Z. Rosenberg, M. M. Segal, M. G. Seidel, D. Smedley, T. Talmy, Y. Thomas, S. A. Wiafe, J. Xian, Z. Yüksel, I. Helbig, C. J. Mungall, M. A. Haendel, and P. N. Robinson. The Human Phenotype Ontology in 2021. *Nucleic acids research*, 49(D1):D1207–D1217, 1 2021.

[11] S. Lee, C. Zhang, M. Arif, Z. Liu, R. Benfeitas, G. Bidkhori, S. Deshmukh, M. Al Shobky, A. Lovric, J. Boren, J. Nielsen, M. Uhlen, and A. Mardinoglu. TCSBN: a database of tissue and cancer specific biological networks. *Nucleic acids research*, 46(D1):D595–D600, 1 2018.

[12] G. Lemaître, F. Nogueira, and C. K. Aridas. Imbalanced-learn: A python toolbox to tackle the curse of imbalanced datasets in machine learning. *The Journal of Machine Learning Research*, 18(1):559–563, 2017.

[13] Y. Li and J. C. Patra. Genome-wide inferring gene-phenotype relationship by walking on the heterogeneous network. *Bioinformatics*, 26(9):1219–1224, 2010.

[14] F. J. Massey. The Kolmogorov-Smirnov Test for Goodness of Fit. *Journal of the American Statistical Association*, 46(253):68–78, 1951.

[15] C. Nachtegael, B. Gravel, A. Dillen, G. Smits, A. Nowé, S. Papadimitriou, and T. Lenaerts. Scaling up oligogenic diseases research with OLIDA: the Oligogenic Diseases Database. *Database*, 2022, 2022.

[16] A. Renaux. BOCK: Bioinformatics networks and Oligogenic Combinations as a Knowledge graph, 2023.

[17] P. Rentzsch, D. Witten, G. M. Cooper, J. Shendure, and M. Kircher. CADD: predicting the deleteriousness of variants throughout the human genome. *Nucleic Acids Research*, 47(D1):D886–D894, 10 2018.

[18] H. A. Shihab, M. F. Rogers, C. Campbell, and T. R. Gaunt. HIPred: an integrative approach to predicting haploinsufficient genes. *Bioinformatics (Oxford, England)*, 33(12):1751–1757, 6 2017.

[19] D. Szklarczyk, A. L. Gable, K. C. Nastou, D. Lyon, R. Kirsch, S. Pyysalo, N. T. Doncheva, M. Legeay, T. Fang, P. Bork, L. J. Jensen, and C. von Mering. The STRING database in 2021: customizable protein-protein networks, and functional characterization of user-uploaded gene/measurement sets. *Nucleic acids research*, 49(D1):D605–D612, 1 2021.

[20] A. Valdeolivas, L. Tichit, C. Navarro, S. Perrin, G. Odelin, N. Levy, P. Cau, E. Remy, and A. Baudot. Random walk with restart on multiplex and heterogeneous biological networks. *Bioinformatics*, 35(3):497–505, 2 2019.

[21] N. Versbraegen, B. Gravel, C. Nachtegael, A. Renaux, E. Verkinderen, A. Nowé, T. Lenaerts, and S. Papadimitriou. Faster and more accurate pathogenic combination predictions with VarCoPP2.0. *BMC Bioinformatics 2023 24:1*, 24(1):1–19, 5 2023.

[22] I. Walsh, D. Fishman, D. Garcia-Gasulla, T. Titma, G. Pollastri, J. Harrow, F. E. Psomopoulos, and S. C. E. Tosatto. DOME: recommendations for supervised machine learning validation in biology. *Nature methods*, 18(10):1122–1127, 2021.
